# Coffee consumption and risk of cancers: a meta-analysis of cohort studies

**DOI:** 10.1186/1471-2407-11-96

**Published:** 2011-03-15

**Authors:** Xiaofeng Yu, Zhijun Bao, Jian Zou, Jie Dong

**Affiliations:** 1 Department of Gastroenterology, Huadong Hospital, Fudan University, Shanghai 200040, PR China

## Abstract

**Background:**

Coffee consumption has been shown to be associated with cancer of various sites in epidemiological studies. However, there is no comprehensive overview of the substantial body of epidemiologic evidence.

**Methods:**

We searched MEDLINE, EMBASE, Science Citation Index Expanded and bibliographies of retrieved articles. Prospective cohort studies were included if they reported relative risks (RRs) and corresponding 95% confidence intervals (CIs) of various cancers with respect to frequency of coffee intake. We did random-effects meta-analyses and meta-regressions of study-specific incremental estimates to determine the risk of cancer associated with 1 cup/day increment of coffee consumption.

**Results:**

59 studies, consisting of 40 independent cohorts, met the inclusion criteria. Compared with individuals who did not or seldom drink coffee per day, the pooled RR of cancer was 0.87 (95% CI, 0.82-0.92) for regular coffee drinkers, 0.89 (0.84-0.93) for low to moderate coffee drinkers, and 0.82 (0.74-0.89) for high drinkers. Overall, an increase in consumption of 1 cup of coffee per day was associated with a 3% reduced risk of cancers (RR, 0.97; 95% CI, 0.96-0.98). In subgroup analyses, we noted that, coffee drinking was associated with a reduced risk of bladder, breast, buccal and pharyngeal, colorectal, endometrial, esophageal, hepatocellular, leukemic, pancreatic, and prostate cancers.

**Conclusions:**

Findings from this meta-analysis suggest that coffee consumption may reduce the total cancer incidence and it also has an inverse association with some type of cancers.

## Background

Coffee is one of the most widely consumed beverages in the world, with a yearly world average consumption of 1.1 kg per capita, which reaches 4.5 kg in industrialized countries[1]. More recently, coffee consumption has been associated with reductions in the risk of several chronic diseases, including type 2 diabetes mellitus, Parkinson's disease and hepatocellular disease[2-4]. Among them, the relationship between coffee drinking and cancer risk holds great interest.

Roasted coffee is a complex mixture of more than a thousand chemicals. Many constituents in it could potentially alter cancer risk through several biological mechanisms. Coffee is the major source of caffeine which has been reported to both stimulate and suppress tumors, depending upon the species and the phase of administration[5]. There are two specific diterpenes in coffee, cafestol and kahweal, which produce biological effects compatible with anticarcinogenic properties, including the induction of phase II enzymes involved in carcinogen detoxification,[6] specific inhibition of the activity of phase I enzyme responsible for carcinogen activation and stimulation of intracellular antioxidant defence mechanisms[7]. Polyphenols are an important ingredient in coffee, such as lignan phytoestrogens and flavonoids and polyphenols are found to exhibit anticarcinogenic properties in several studies[8]. Caffeic acid has the ability to inhibit DNA methylation in cultured human cancer cells and is associated with inactivation of various pathways involved in the tumorigenic process, including cell cycle regulation, inflammatory and stress response and apoptosis[9]. Coffee is also a major source of the chlorogenic acid that contributes to its antioxidant effect. Intake of chlorogenic acid has been shown to reduce glucose concentrations in rats and intake of quinides, degradation products of chlorogenic acid, increases insulin sensitivity[10]. Chronic hyperinsulinemia and insulin resistance are confirmed markers of high risk for some cancer sites[11].

Over the last 4 decades, a number of epidemiologic studies (over 500 papers) have estimated the associations between coffee consumption and cancer occurrence at various sites. However, their results were inconsistent. In 2007, the World Cancer Research Fund (WCRF) conducted a comprehensive analysis of diet and cancer, using a more standardized approach to review the evidence. This report addressed the significant relationships between coffee and risk of pancreatic and kidney cancer[5]. In fact, epidemiologic studies have been published relating coffee intake to cancers of 11 different organ sites. Data from case-control studies may be subject to recall bias with respect to coffee consumption and selection bias with respect to the control group. Additional prospective cohort studies excluding those biases would be more useful to see coffee-cancer associations. We therefore systematically reviewed and performed a meta-analysis of prospective cohort studies to quantitatively assess the association between coffee intake and cancer risk in human. Because of the high consumption of coffee, even small effects on cancer occurrence in persons could have a large impact on public health.

## Methods

### Literature search

We searched the electronic databases MEDLINE (1966 to March, 2010), EMBASE (1985 to March, 2010), and Science Citation Index Expanded (1945 to March, 2010), using the Medical Subject Heading (MeSH) term *coffee *combined with *cancer *or *neoplasm *or *carcinoma*. Furthermore, we reviewed reference lists of retrieved articles to search for more studies. Only those that were published as full-length articles and in English were considered.

### Inclusion and exclusion criteria

For inclusion, studies had to fulfil the following criteria: have a prospective cohort design; report relative risks or hazard ratios and their corresponding 95% CIs (or data to calculate them) of cancer relating to every category of coffee intake; and provide the frequency of coffee consumption. Studies were excluded if: case-control design was used; mixed beverage was reported, in which the effect of coffee could not be separated; only surrogate nutrients of coffee were reported; no categories of coffee intake were reported that could not allow for adequate classification of intake. If multiple published reports from a same study cohort were available, we included only the one with the most detailed information for both outcome and coffee consumption.

### Data extraction

Data were extracted independently by two investigators (Yu and Bao) according to the meta-analysis of observation studies in epidemiology (MOOSE) guidelines,[12] and discrepancies were resolved by discussion with a third investigator (Zou). For each study, the following information was extracted: first author's last name; year of publication; country of origin; follow-up period; number of subjects and cases; age at baseline; cancer sites; category amounts of coffee intake; outcome assessment; relative risks or hazard ratios of cancer and corresponding 95% CIs for every category of coffee intake; and covariates adjusted in the statistical analysis.

### Statistical analysis

The measures of interest were the RR and the corresponding 95% CIs for the included cohort studies. When RRs were not available in the published article, they were computed from the exposure distributions. Because various studies used different measurement units for coffee consumption, we converted these into cups per day as a standard measure. If coffee consumption was indicated in milliliters, we assumed 125 mL as approximately equivalent to 1 cup.

We computed the summary RR for coffee drinkers versus nondrinkers and for different levels of consumption by giving each study-specific RR a weight that was proportional to its precision (ie, the inverse of the variance derived, when necessary, from the reported 95% CIs). To estimate the summary RR for various levels of coffee consumption, we first calculated the study-specific estimate separately for low to moderate consumption and high consumption. For various cancer sites, we performed stratified analysis on cancer types which had more than two cohorts.

Statistical heterogeneity among studies was estimated using Q and I^2 ^statistics. For the Q statistic, heterogeneity was considered present for P < 0.1. We pooled the study-specific estimates using both the fixed effect model and the random effect model proposed by DerSimonian and Laird; when a significant heterogeneity was found, the random effect model results were presented. A sensitivity analysis was also conducted, in which 1 study at a time was removed and the others analyzed to estimate whether the results could have been affected markedly by a single study.

For dose-response analysis, we used the method proposed by Greenland and Longnecker[13] to estimate study-specific slopes from the correlated natural logarithm of the RR across categories of coffee consumption, assigning to each class the dose corresponding to the midpoint of upper and lower boundaries. The highest, open-ended category was assumed to have the same amplitude of consumption as the preceding category[14]. Then the summary RR for cancer risk with 1 cup/day increment of coffee consumption was obtained by pooling the study-specific slopes, using the inverse of the corresponding variances as weights.

Finally, publication bias was evaluated through funnel plot visual analysis and with the Begg's and Egger's tests. P < 0.05 was considered statistically significant. All statistical analyses were performed with STATA (version 9.0; Stata Corp, College Station, TX).

## Results

**Figure 1:**
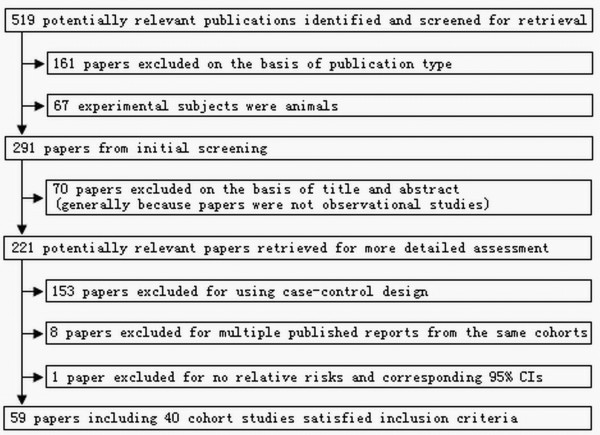
**Flow diagram of search strategy and study selection**.

Using the predefined search strategy, we identified 59 publications, 40 prospective cohort studies, (Figure 1) including 2,179,126 participants and 34,177 incident cases of cancer with an average follow-up of 14.3 years, which were eligible for inclusion in the meta-analysis[15-73]. The characteristics of the included studies are summarized in Additional file: 1. Initial agreement between the two reviewers on whether a study was eligible for inclusion occurred in 207/221 manuscripts (93.7%; κ = 0.874). Of the 40 cohorts included in the meta-analysis, 13 were conducted in Europe (Norway, Denmark, Sweden, France, Finland, and Netherlands), 15 in North America (Canada and the United States), and 12 in Asia (Japan and Singapore).

The estimated RRs of various cancer sites for coffee drinkers vs non/lowest drinkers was 0.87 (95% CI, 0.82-0.92). There was significant heterogeneity across the studies (*Q *= 178.1, *P *< 0.001, *I*^2 ^= 78.1%). The summary RR was 0.89 (95% CI, 0.84-0.93) for low to moderate coffee consumption, with a significant heterogeneity between studies (*Q *= 95.78, *P *< 0.001, *I*^2 ^= 61.4%). That for high consumption of coffee was 0.82 (95% CI, 0.74-0.89), also with a significant heterogeneity between studies (*Q *= 114.71, *P *< 0.001, *I*^2 ^= 67.7%).

Various sources of heterogeneity likely exist due to international differences in coffee consumption (e.g., coffee type, serving size, or brewing method) in this analysis. To examine the magnitude of the combined RR in each stratum and its respective test of heterogeneity, we conducted subgroup analyses by gender, cancer sites, and geographic regions. The summary RR was 0.88 (95% CI, 0.78-0.98) for men and 0.87 (95% CI, 0.82-0.93) for women combining all studies. There was a significant heterogeneity for men (*Q *= 118.27, *P *< 0.001, *I*^2 ^= 84.8%) and for women (*Q *= 90.61, *P *< 0.001, *I*^2 ^= 75.7%).

**Figure 2:**
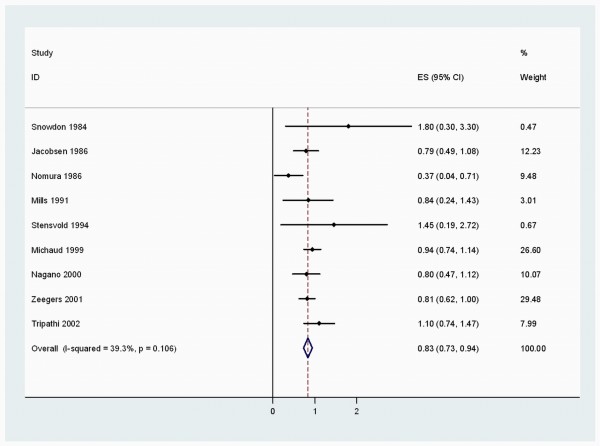
**Summary RRs of bladder cancer for coffee drinkers versus non/lowest drinkers from included studies**. Squares represent study-specific relative risk estimates (size of the square reflects the study-specific statistical weight, that is, the inverse of the variance); *horizontal lines *represent 95% CIs; *diamonds *represent summary relative risk estimates with corresponding 95% CIs.

**Figure 3:**
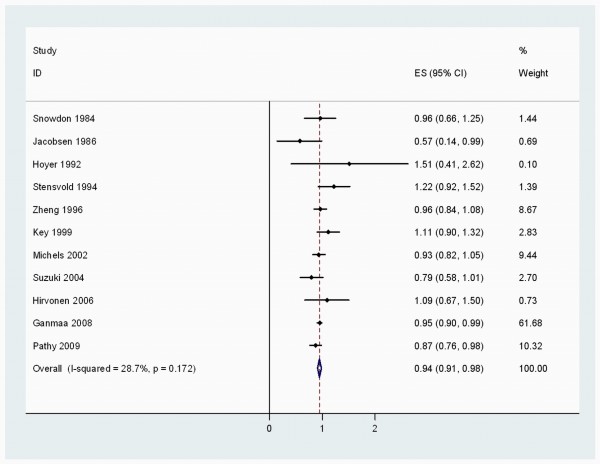
**Summary RRs of breast cancer for low to moderate coffee drinkers versus non/lowest drinkers from included studies**. *Squares *represent study-specific relative risk estimates (size of the square reflects the study-specific statistical weight, that is, the inverse of the variance); *horizontal lines *represent 95% CIs; *diamonds *represent summary relative risk estimates with corresponding 95% CIs.

**Figure 4:**
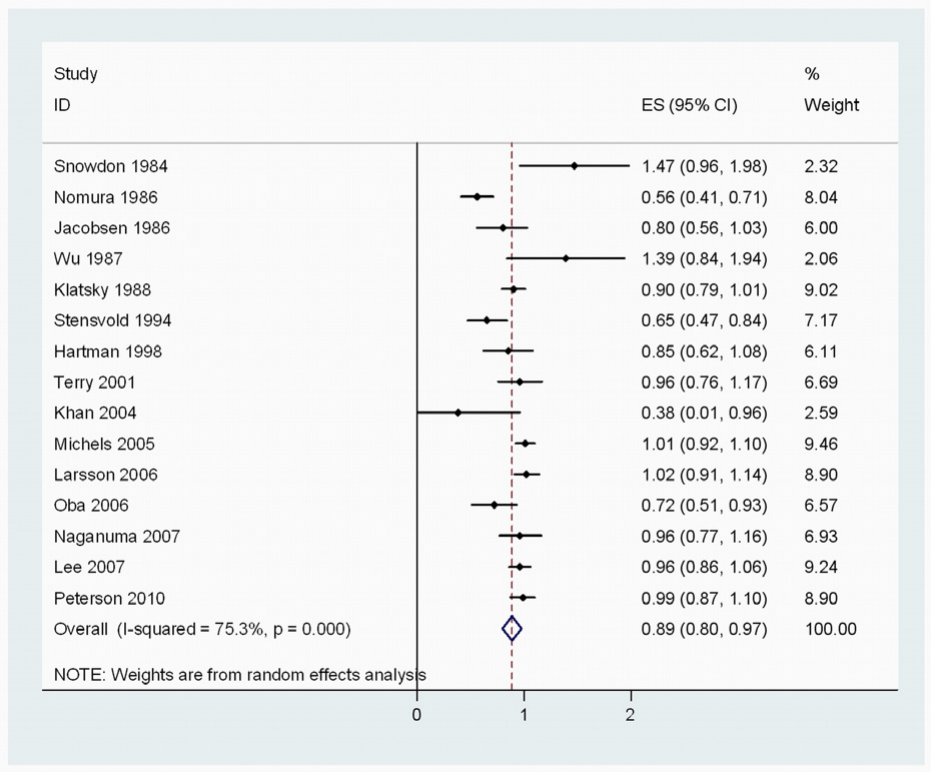
**Summary RRs of colorectal cancer for high coffee drinkers versus non/lowest drinkers from included studies**. *Squares *represent study-specific relative risk estimates (size of the square reflects the study-specific statistical weight, that is, the inverse of the variance); *horizontal lines *represent 95% CIs; *diamonds *represent summary relative risk estimates with corresponding 95% CIs.

**Figure 5:**
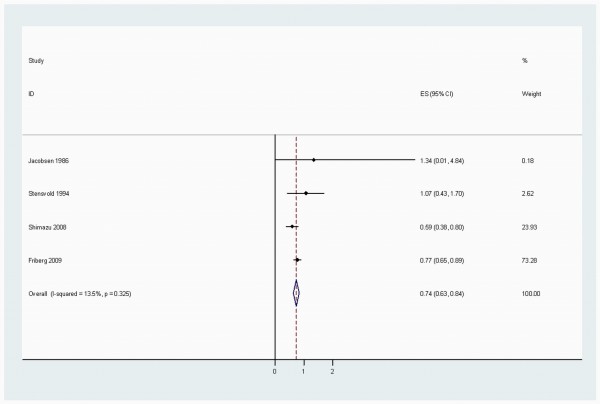
**Summary RRs of endometrial cancer for high coffee drinkers versus non/lowest drinkers from included studies**. *Squares *represent study-specific relative risk estimates (size of the square reflects the study-specific statistical weight, that is, the inverse of the variance); *horizontal lines *represent 95% CIs; *diamonds *represent summary relative risk estimates with corresponding 95% CIs.

**Figure 6:**
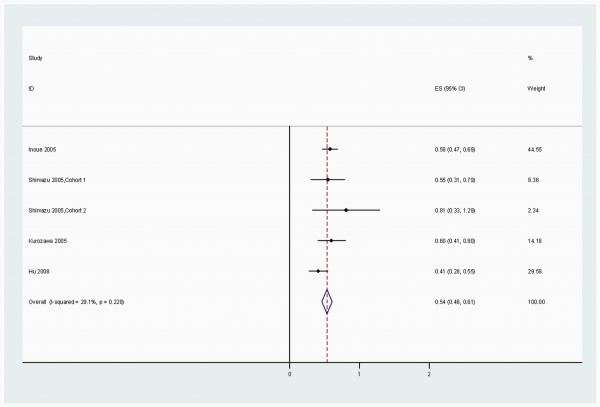
**Summary RRs of hepatocellular cancer for high coffee drinkers versus non/lowest drinkers from included studies**. *Squares *represent study-specific relative risk estimates (size of the square reflects the study-specific statistical weight, that is, the inverse of the variance); *horizontal lines *represent 95% CIs; *diamonds *represent summary relative risk estimates with corresponding 95% CIs.

**Figure 7:**
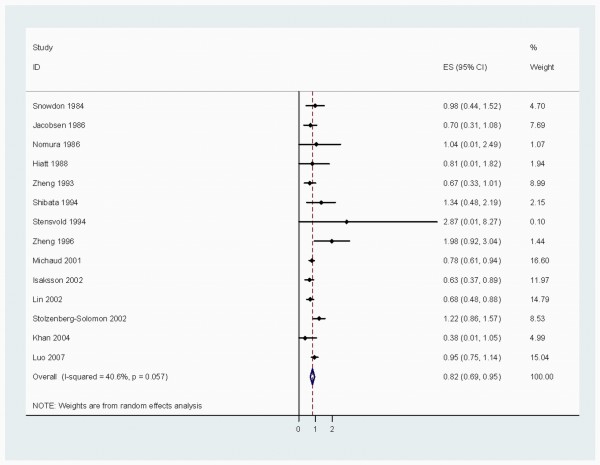
**Summary RRs of pancreatic cancer for high coffee drinkers versus non/lowest drinkers from included studies**. *Squares *represent study-specific relative risk estimates (size of the square reflects the study-specific statistical weight, that is, the inverse of the variance); *horizontal lines *represent 95% CIs; *diamonds *represent summary relative risk estimates with corresponding 95% CIs.

**Figure 8:**
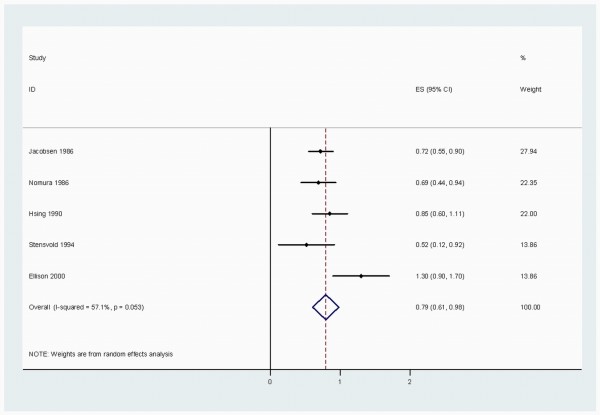
**Summary RRs of prostate cancer for high coffee drinkers versus non/lowest drinkers from included studies**. *Squares *represent study-specific relative risk estimates (size of the square reflects the study-specific statistical weight, that is, the inverse of the variance); *horizontal lines *represent 95% CIs; *diamonds *represent summary relative risk estimates with corresponding 95% CIs.

When stratified by cancer sites, we noted that, coffee consumption was inversely associated with bladder (RR 0.83 (95% CI, 0.73-0.94)) (Figure 2), breast 0.94 (0.91-0.98) (Figure 3), buccal and pharyngeal 0.49 (0.29-0.70), colorectal 0.89 (0.80-0.97) (Figure 4), endometrial 0.74 (0.63-0.84) (Figure 5), esophageal 0.55 (0.37-0.74), hepatocellular 0.54 (0.46-0.61) (Figure 6), leukemic 0.64 (0.51-0.77), pancreatic 0.82 (0.69-0.95) (Figure 7), and prostate 0.79 (0.61-0.98) (Figure 8) cancers. There appeared to be no association with stomach, lung, nonmelanoma, ovarian, or kidney cancer. The summary RR for an increment of 1 cup of coffee per day was 0.97 (95% CI, 0.96-0.98) for all studies combined. The pooled RR for various cancer sites and incremental estimates for 1 cup/day increment of coffee consumption and their heterogeneity are listed in Additional file 2: Table S2

Associations were also similar in studies from North America, Europe, and the Asia-Pacific region. The RR was 0.92 (95% CI, 0.86-0.98) when considering 15 studies conducted in North America, 0.85 (95% CI, 0.72-0.98) for 13 studies from Europe and 0.82 (95% CI, 0.74-0.90) for 12 Asian studies. No significant differences by sex and cancer-site were found.

**Figure 9:**
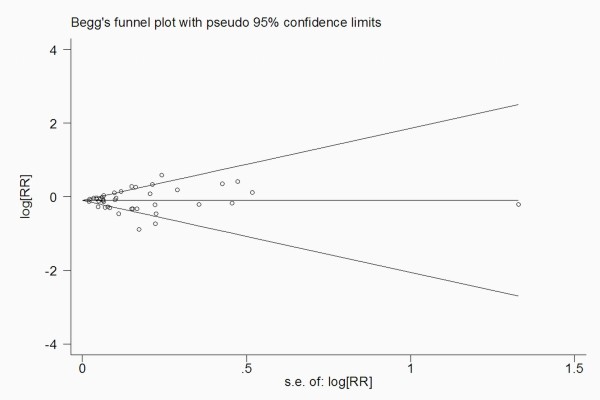
**Publication bias in the studies**. Begg's funnel plot indicating no publication bias in the studies included in this meta-analysis. No indication of publication bias was noted from both visualization of funnel plot and Egger's test.

There was no indication of publication bias from either visualization of the funnel plot or Egger's (*P = 0*.793) and Begg's (*P = 0*.981) (Figure 9) tests. A sensitivity analysis, in which 1 study was removed at a time, was performed to evaluate the stability of the results. This analysis confirmed the stability of our results.

## Discussion

Coffee can potentially impact the etiology of cancer of various sites along multiple pathways, ranging from carcinogenesis to cellular apoptosis. For most cancer sites, there is a significant amount of evidence showing no detrimental effect of consumption of up to 6 cups of coffee per day in relation to cancer occurrence. Through the meta-analysis of cohort studies, we found that compared with individuals who did not or seldom drink coffee per day, regular coffee drinkers had lower cancer occurrence, particularly for high drinkers. Overall, an increase in consumption of 1 cup of coffee per day was associated with a 3% reduced risk of cancers. The evidence presented above suggests that coffee intake might reduce cancer occurrence in humans.

A significant amount of literature exists on relationships between coffee consumption and human cancer occurrence at 11 organ sites. It has been confirmed that coffee consumption is associated with a reduced risk of hepatocellular, kidney, and to a lesser extent, premenopausal breast and colorectal cancers, while it is unrelated to prostate, pancreas and ovary cancers.n subgroup analyses, we note that, for bladder, breast, buccal and pharyngeal, colorectal, endometrial, esophageal, hepatocellular, leukemic, pancreatic, and prostate cancers, there appears to be an inverse association.

Over the past two decades, many studies have been carried out after the early warning in the early 1980 s that coffee consumption was related to pancreatic cancer risk. These investigations yield inconsistent results. Since the WCRF report, Luo et al[59] studied the association between the drinking coffee and the risk of pancreatic cancer in a large population-based cohort study in Japan and concluded there was no increased risk of pancreatic cancer with coffee intake. A reduced risk was apparent among men who drank at least 3 cups of coffee per day. After a pooled analysis of 14 cohort studies, we found that coffee consumption had a significantly inverse association with the risk of pancreatic cancer.

Among investigations that have addressed the association between coffee consumption and the risk of kidney cancer, a pooled analysis of 13 cohort studies found that, coffee consumption was associated, but not significantly, with a lower risk of kidney cancer[74]. Among the participants in the Nurses' Health Study and the Health Professionals Follow up Study, no association was seen between coffee intakes and risk of kidney cancer[63]. However, this conclusion is not confirmed by our results. There was a protective effect on men who drink coffee and for high coffee drinkers. Coffee consumption may reduce kidney cancer risk because caffeine has a diuretic effect by blocking anti-diuretic hormone and antioxidants in coffee alleviate oxidative damage to DNA, proteins and other molecules. Moreover, coffee consumption may reduce the risk of kidney cancer by improving insulin sensitivity[75].

Colorectal cancer is one of the most common cancers worldwide. It has been suggested that coffee is a protective factor against colorectal cancer through its carcinogenic constituents, cafestol and kahweal and its ability to induce excretion of bile acids and neutral sterols into the colon[76]. Moreover, coffee might decrease colorectal cancer risk by increasing large bowel mobility in the rectosigmoid region, while caffeine has been shown to inhibit colon cancer cell growth[77]. A meta-analysis of prospective cohort studies on colorectal cancer and coffee consumption was completed and published in 2009[78]. The result of it showed no significant effect of coffee consumption on colorectal cancer risk. However, in our meta-analysis, 15 cohorts were identified from Japan, Norway, Finland, Singapore, Sweden, and the United States. We found that coffee consumption had an inverse association with the risk of colorectal cancer.

Preliminary results from the Nurses Health Study suggested a weak inverse association between caffeine intake and the risk of breast cancer[44]. A Norwegian cohort of 14,593 women who drank ≥5 cups of coffee per day experienced a statistically significant 50% decrease in breast cancer risk compared to those who drank ≤2 cups[79]. A meta-analysis of 9 cohort and 9 case-control studies found a borderline significant influence of highest coffee consumption on the risk of breast cancer. The results of our meta-analysis also confirmed the former conclusion and showed coffee drinking had an inverse association with breast cancer. We also observed a reduction of 26% in the risk of endometrial cancer among coffee drinkers, compared with nondrinkers, and of >30% among heavy coffee drinkers.

In addition, higher intake of caffeine and caffeine-containing beverages has been positively associated with sex hormone binding globulin and inversely associated with bioavailable testosterone[80]. These hormonal changes may favorably influence breast or endometrial cancer risk. Coffee consumption was also shown to be associated with increased ratio of plasma 2-hydroxyestrone to 16-alphahydroxyestrone, a predictor of lower breast cancer risk[81].

A meta-analysis, including 6 case-control and 4 cohort studies reported a statistically significant 41% reduction in the hepatocellular cancer risk among coffee drinkers compared with never drinkers, with similar results from case-control and prospective studies[82]. Another meta-analysis of 4 cohort and 5 case-control studies found that an increased coffee consumption is associated with a reduced risk of hepatocellular cancer, both among individuals with and without a history of hepatocellular disease[83]. Our meta-analysis including 5 cohort studies also suggested a significant inverse relation between coffee intake and hepatocellular cancer.

A protective effect of coffee consumption on hepatocellular cancer is biologically plausible. Coffee contains large amounts of antioxidants, such as chlorogenic acids, and experimental studies in animals have demonstrated an inhibitory effect of coffee and chlorogenic acids on hepatocellular carcinogenesis[84]. In one animal study, caffeine levels of coffee extracts were inversely related to hepatocellular injury[85]. A population-based case-control study in the United States showed that higher intake of coffee, and especially caffeine, was associated with a lower prevalence of abnormal alanine aminotransferase activity[86]. In addition, some studies have reported an inverse association between coffee consumption and risk of hepatocellular cirrhosis, which is strongly related to HCC[87].

Coffee consumption and cancer of the urinary track was systematically reviewed in 2001[88]. We incorporated data on adjusted summary RRs from 9 cohort studies and found coffee to be inversely associated with bladder cancer in men, whereas the trend was not seen in women. The Lutheran Brotherhood Cohort study found coffee consumption unrelated to prostate cancer risk[60]. But we found that the summary RR of prostate cancer was 0.79 for coffee drinkers vs nondrinkers.

Some limitations of this meta-analysis should be acknowledged. First, as in all observational studies of diet and disease, the possibility of bias and confounding can not be excluded (for some subjects may have modified their coffee drinking habit after the baseline assessment). However, cohort studies, which are less susceptible to bias because of the prospective design, also showed an inverse association between coffee consumption and risk of cancer, suggesting that the finding is not likely attributable to recall and selection bias. Individual studies may have failed to adjust for potential known or unknown confounders. Second, our results are likely to be affected by some misclassification of coffee consumption. Coffee exposure is mostly assessed regarding the number of cups of coffee consumed daily, weekly or monthly. However, most of the studies included in our meta-analysis did not provide information on coffee type, serving size, or brewing method. Serving sizes and brewing methods for coffee can vary substantially within and between countries. The size of standard coffee cups is larger in the United States compared with that in Europe or Japan, and the difference in the strength of coffee brew may compensate for the different serving size between countries[89]. Third, we extracted the risk estimates that reflected the greatest degree of the control potential confounders, the results based on the adjustment for different confounders were probably different from those based on standardized adjustments. Finally, only published studies were included in our meta-analysis. Therefore, publication bias may have occurred although no publication bias was indicated from both visualization of the funnel plot and Egger's test.

## Conclusions

All in all, our meta-analysis including 40 prospective cohort studies confirmed that coffee drinking have no harmful effect. Instead, coffee consumption is inversely associated with the risk of bladder, breast, buccal cavity and pharynx, colorectum, endometrium, esophagus, hepatocellular, leukemia, pancreas, and prostate cancers.

## Competing interests

The authors declare that they have no competing interests.

## Authors' contributions

XY and JZ conceived the study. Data was acquired independently by XY and ZB. JD and JZ undertook data analysis and interpretation. JZ prepared the manuscript with contributions from all co-authors. All authors read and approved the final manuscript

## Pre-publication history

The pre-publication history for this paper can be accessed here:

http://www.biomedcentral.com/1471-2407/11/96/prepub

## Supplementary Material

Additional file 1 **Table S1**. Summary characteristics of studies included in the meta-analysis. Click here for file

Additional file 2 **Table S2**. The summary RR for various cancer sites or different geographic regions and incremental estimates for 1 cup/day increment of coffee consumption. Click here for file
