# Supplementary material for: Coffee consumption and risk of cancers: a meta-analysis of cohort studies
Source: BMC Cancer. 2011 Mar 15;11:96. doi: 10.1186/1471-2407-11-96 (PMC3066123; doi:10.1186/1471-2407-11-96)
Supplement: Additional file 1 — Table S1. Summary characteristics of studies included in the meta-analysis. [file 1471-2407-11-96-S1.DOC]

Table S1 Summary characteristics of studies included in the meta-analysis.

|  |  | Follow-up | Study | No.of | | Coffee | Relative risk |  |
| --- | --- | --- | --- | --- | --- | --- | --- | --- |
| Study | Country | period | subjects | cases | Cancer site | consumption | (95% CI) | Adjustments |
| Inoue et al^15^ | Japan | 1990-2001 | 90,452 | 334 | Hepatocellular | Almost never | 1.00(reference) | Age, sex, study area, |
| 2005 |  |  | 43,109 M | 250 M |  | 1-2 days/week | 0.75(0.56-1.01) | smoking, intakes of alcohol, |
|  |  |  | 47,343 F | 84 F |  | 3-4 days/week | 0.79(0.55-1.14) | green tea, green vegetables |
|  |  |  | Aged 40-69 y |  |  | 1-2 cups/day | 0.52(0.38-0.73) |  |
|  |  |  |  |  |  | 3-4 cups/day | 0.48(0.28-0.83) |  |
|  |  |  |  |  |  | ≥5 cups/day | 0.24(0.08-0.77) |  |
| Shimazu et al^16^ | Japan | 1984-1992 | 22,404 | 70 | Hepatocellular | Nondrinkers | 1.00(reference) | Age, sex, smoking, |
| 2005,Cohort 1 |  |  | 10,588 M | 50 M |  | Occasionally | 0.56(0.33-0.97) | alcohol intake, |
|  |  |  | 11,816 F | 20 F |  | ≥1 cup/day | 0.53(0.28-1.00) | history of hepatocellular disease |
|  |  |  | Aged ≥40 y |  |  |  |  |  |
| Shimazu et al^16^ | Japan | 1990-1997 | 38,703 | 47 | Hepatocellular | Nondrinkers | 1.00(reference) | Age, sex, smoking, |
| 2005,Cohort 2 |  |  | 18,869 M | 41 M |  | Occasionally | 1.05(0.52-2.16) | , |
|  |  |  | 19,834 F | 6 F |  | ≥1 cup/day | 0.68(0.31-1.51) | history of hepatocellular disease |
|  |  |  | Aged 40-64 y |  |  |  |  |  |
| Kurozawa et al^17^ | Japan | 1988-1999 | 83,966 | 258 | Hepatocellular | Nondrinkers | 1.00(reference) | Age, sex, education, |
| 2005 |  |  | 35,179 M | 178 M |  | ＜1 cup/day | 0.83(0.54-1.25) | smoking, alcohol intake, |
|  |  |  | 48,787 F | 80 F |  | ≥1 cup/day | 0.50(0.31-0.79) | history of diabetes or hepatocellular disease |
|  |  |  | Aged 40-79 y |  |  |  |  |  |
| Hu et al^18^ | Finland | 1972-2006 | 60,323 | 128 | Hepatocellular | 0-1 cups/day | 1.00(reference) | Age, sex, education, |
| 2008 |  |  | 29,286 M | 82 M |  | 2-3 cups/day | 0.66(0.37-1.16) | smoking, alcohol consumption, |
|  |  |  | 31,037 F | 46 F |  | 4-5 cups/day | 0.44(0.25-0.77) | during follow-up, BMI |
|  |  |  | Aged 25-74 y |  |  | 6-7 cups/day | 0.38(0.21-0.69) | history of diabetes or hepatocellular disease |
|  |  |  |  |  |  | ≥8 cups/day | 0.32(0.16-0.62) |  |
| Shimazu et al^19^ | Japan | 1990-2005 | 53,724 F | 117 | Uterus | ≤2 days/week | 1.00(reference) | Age, study center, BMI, smoking status, |
| 2008 |  |  | Aged 40–69 y | |  | 3-4 days/week | 0.97(0.56-1.68) | menopausal status, age at menopause, use |
|  |  |  |  |  |  | 1-2 cups/day | 0.61(0.39-0.97) | of exogenous hormones, beef consumption, |
|  |  |  |  |  |  | ≥3 cups/day | 0.38(0.16-0.91) | pork consumption, green tea consumption, |
|  |  |  |  |  |  |  |  | green vegetable consumption |
| Friberg et al^20^ | Sweden | 1987-2007 | 60,634 F | 677 | Uterus | ≤1 cup/day | 1.00(reference) | Age in months, |
| 2009 |  |  | Aged 40-76 y |  |  | 2-3 cups/day | 0.78(0.64-0.95) | BMI, smoking |
|  |  |  |  |  |  | ≥4 cups/day | 0.75(0.58-0.97) |  |
| Larsson et al^21^ | Sweden | 1987-2004 | 61,057 F | 301 | Ovary | ＜1 cup/day | 1.00(reference) | Age, BMI, education, parity, |
| 2005 |  |  | Aged 40-76 y |  |  | 1 cup/day | 1.13(0.69-1.86) | oral contraceptive use, |
|  |  |  |  |  |  | 2-3 cups/day | 0.97(0.62-1.51) | intakes of total energy, |
|  |  |  |  |  |  | ≥4 cups/day | 1.07(0.64-1.79) | fruit, vegetables, milk, tea |
| Silvera et al^22^ | Canada | 1980-2000 | 48,776 F | 264 | Ovary | None | 1.00(reference) | Age, BMI, education, smoking history, |
| 2007 |  |  | Aged 40-59 y |  |  | 0-1 cups/day | 1.18(0.76-1.83) | pack-years of smoking, alcohol intake, |
|  |  |  |  |  |  | 2-3 cups/day | 1.36(0.86-2.15) | menopausal status, oral contraceptive use, |
|  |  |  |  |  |  | ≥4 cups/day | 1.62(0.95-2.75) | parity, energy intake, lactose intake, |
|  |  |  |  |  |  |  |  | participation in vigorous physical |
|  |  |  |  |  |  |  |  | activity, study center, randomization group |
| Steevens et al^23^ | Netherlands | 1986-1999 | 62,573 F | 280 | Ovary | 0-1 cups/day | 0.73(0.41-1.31) | Age, parity, |
| 2007 |  |  |  |  |  | 1-3 cups/day | 1.00(reference) | cigarette smoking, |
|  |  |  |  |  |  | 3-5 cups/day | 1.00(0.74-1.35) | oral contraceptive use |
|  |  |  |  |  |  | ≥5 cups/day | 1.08(0.75-1.57) |  |
| Tworoger et al^24^ | United | 1980-2004 | 80,253 F | 507 | Ovary | None | 1.00(reference) | Age, parity, BMI, |
| 2008 | States |  |  |  |  | ＞0-6 cups/week | 0.84(0.62-1.14) | smoking status, |
|  |  |  |  |  |  | 1 cups/day | 1.01(0.75-1.36) | tubal ligation, |
|  |  |  |  |  |  | 2 cups/day | 0.87(0.65-1.16) | oral contraceptive use, |
|  |  |  |  |  |  | ≥3 cups/day | 0.75(0.55-1.02) | postmenopausal hormone use, |
| van Loon et al^25^ | Netherlands | 1986-1990 | 58,279 M | 162 | Stomach | ≤3 cups/day | 1.0(reference) |  |
| 1998 |  |  | Aged 55–69 y | |  | > 4 cups/day | 1.5(0.95-2.36) |  |
| Galanis et al^26^ | United | 1975-1994 | 11,907 | 108 | Stomach | none | 1.0(reference) | Age, sex, education, |
| 1998 | States |  | 5610 M | 64 M |  | 1 cup/day | 1.8(1.0-3.2) | Japanese place of birth, |
|  |  |  | 6297 F | 44 F |  | ≥2 cup/day | 1.8(1.0-3.3) | smoking and alcohol intake(only in men) |
|  |  |  | Aged ≥18 y |  |  |  |  |  |
| Tsubono et al^27^ | Japan | 1984-1992 | 26,311 | 419 | Stomach | never | 1.0(reference) | Sex, age, tea, smoking, |
| 2001 |  |  | 11,902 M | 296 M |  | occasionally | 0.9(0.6-1.2) | consumption of alcohol, rice, |
|  |  |  | 14,409 F | 123 F |  | 1-2 cups/day | 0.8(0.5-1.0) | meat, vegetables, fruits, bean-past |
|  |  |  | Aged ≥40 y |  |  | ≥3 cups/day | 1.0(0.6-1.6) | soup, type of health insurance |
| Larsson et al^28^ | Sweden | 1987-2005 | 61,433 F | 160 | Stomach | ≤1 cup/day | 1.00(reference) | Age,time period, education, |
| 2006 |  |  | Aged 40–76 y | |  | 2-3 cups/day | 1.49(0.97-2.27) | alcohol intake, tea consumption |
|  |  |  |  |  |  | ≥4 cups/day | 1.86(1.07-3.25) |  |
| Wu et al^29^ | United | 1981-1985 | 11,644 | 126 | Colorectum | 0-1 cups/d | 1.00(reference) | Age |
| 1987 | States |  |  | 58 M |  | 2-3 cup/d | 1.41(0.76-2.06) |  |
|  |  |  |  | 68 F |  | ≥4 cups/d | 1.33(0.31-2.35) |  |
| Klatsky et al^30^ | United | 1978-1984 | 106,203 | 269 | Colorectum | Continuous | 1.00(reference) | Age, sex, alcohol, smoking, BMI, |
| 1988 | States |  |  | 203 CC | | variable | 0.90(0.79-1.01) | race, education, serum cholesterol |
|  |  |  |  | 66 RC |  |  |  |  |
| Hartman et al^31^ | Finland | 1985-1993 | 27,111 M | 185 | Colorectum | ≤4 cups/d | 1.00(reference) | Age, BMI, physical activity, |
| 1998 |  |  | Aged 50-69 y | 106 CC | | >4 cups/d | 0.90(0.57-1.22) | intervention group, |
|  |  |  |  | 79 RC |  | >6 cups/d | 0.79(0.47-1.12) | calcium, tea, |
|  |  |  |  |  |  |  |  | serum cholesterol(for RC) |
| Terry et al^32^ | Sweden | 1987-1998 | 61,463 F | 460 | Colorectum | <1 cup/d | 1.00(reference) | Age, alcohol, BMI, education, |
| 2001 |  |  | Aged 40-74 y | 291 CC | | 1 cup/d | 0.96(0.66-1.40) | calories, red meat, fat, fiber, |
|  |  |  |  | 159 RC | | 2-3 cup/d | 0.93(0.67-1.29) | calcium, folic acid, |
|  |  |  |  |  |  | ≥4 cups/d | 1.04(0.70-1.54) | vitamin C, vitamin D |
| Michels et al^33^ | United | 1980-1998 | 133,893 | 1438 | Colorectum | never | 1.00(reference) | Age, BMI, height, alcohol, smoking, |
| 2005 | States |  | 46,099 M | 1177 CC | | 1/2 cup/d | 1.05(0.88-1.26) | physical activity, red meat, |
|  |  |  | Aged 40-75 y | 261 RC | | 1 cup/d | 0.99(0.82-1.18) | aspirin use, vitamin supplement, |
|  |  |  | 87,794 F |  |  | 2-3 cup/d | 1.02(0.87-1.19) | calories, menopausal status, |
|  |  |  | Aged 34-59 y |  |  | 4-5 cup/d | 0.98(0.80-1.21) | postmenopausal hormone use, |
|  |  |  |  |  |  | >5 cups/d | 0.98(0.69-1.38) | family history of CRC, sigmoidoscopy |
| Larsson et al^34^ | Sweden | 1998-2004 | 81,922 | 723 | Colorectum | <1 cup/d | 1.00(reference) | Age, BMI, smoking, physical activity, |
| 2006 |  |  | 45,306 M | 469 CC | | 1 cup/d | 1.2(0.87-1.65) | family history of CRC and diabetes, |
|  |  |  | Aged 45–79 y | 256 RC | | 2-3 cup/d | 1.13(0.87-1.48) | aspirin use, multivitamin use, milk, |
|  |  |  | 36,616 F |  |  | 4-5 cup/d | 1.15(0.86-1.53) | calories, red meat, fruits, vegetables, |
|  |  |  | Aged 51–87 y | |  | ≥6 cups/d | 1.06(0.74-1.52) | for women post menopausal hormone use |
| Oba et al^35^ | Japan | 1993-2000 | 30,221 | 213 | Colorectum | <1 cup/month | 1.00(reference) | Age, BMI, height, alcohol, smoking, |
| 2006 |  |  | 13,894 M | 111 M |  | 1 cup/month- | 1.00(0.66-1.33) | physical activity, black/green tea |
|  |  |  | 16,327 F | 102 F |  |  |  |  |
|  |  |  | Aged ≥35 y |  |  | ≥1 cup/d | 0.54(0.28-0.81) |  |
|  |  |  |  |  |  |  |  |  |
| Naganuma et al^36^ | Japan | 1990-2001 | 38,701 | 457 | Colorectum | never | 1.00(reference) | Age, sex, BMI, alcohol, smoking, education, |
| 2007 |  |  | 18,867 M | 284 M |  | occasionally | 1.14(0.87-1.46) | walking time, family history of CRC, |
|  |  |  | 19,834 F | 173 F |  | 1-2 cups/d | 0.98(0.74-1.32) | calories, fruits, vegetables, meat, black/ |
|  |  |  | Aged 40-64 y | 281 CC | | ≥3 cups/d | 0.95(0.65-1.39) | green tea, for women menopausal status, |
|  |  |  |  | 180 RC | |  |  | age at menarche, age at first dehepatocellulary, |
|  |  |  |  |  |  |  |  | numbers of pregnancies and dehepatocellularies |
| Lee et al^37^ | Japan | 1990-2002 | 96,162 | 1163 | Colorectum | almost never | 1.00(reference) | Age, BMI, alcohol, smoking, |
| 2007 |  |  | 46,023 M | 726 M |  | <1 cup/d | 0.94(0.79-1.10) | physical activity, study area, |
|  |  |  | 50,139 F | 437 F |  | 1-2 cup/d | 1.00(0.79-1.14) | family history of CRC, beef, pork, |
|  |  |  |  | 763 CC | | ≥3 cups/d | 0.92(0.67-1.12) | green vegetables, black/green tea, |
|  |  |  |  | 400 RC | |  |  | Chinese tea |
| Peterson et al^38^ | Singapore | 1993-2005 | 61,321 | 961 | Colorectum | <1 cup/d | 1.00(reference) | age, gender, BMI, dialect group, |
| 2010 |  |  | Aged 45-74 y | 591 CC | | 1 cup/d | 1.02(0.86-1.18) | year of recruitment, level of education, |
|  |  |  |  | 370 RC | | ≥2 cups/d | 0.95(0.79-1.11) | cigarette smoking, alcohol consumption, |
|  |  |  |  |  |  |  |  | physical activity, history of diabetes, |
|  |  |  |  |  |  |  |  | family history of CRC, green tea intake |
| Hoyer et al^39^ | Denmark | 1964-1986 | 5,207 F | 51 | Breast | 0-2 cups/d | 1.0(reference) | Social class, age at menarche, |
| 1992 |  |  | Aged 30-80 y |  |  | 3-6 cups/d | 1.4(0.6-3.4) | menopause status, height, smoking status, |
|  |  |  |  |  |  | ≥7 cups/d | 1.7(0.7-4.3) | number of full-term pregnancies, |
|  |  |  |  |  |  |  |  | weight, BMI, alcohol consumption |
| Key et al^40^ | Japan | 1969-1993 | 34,759 F | 344 | Breast | ≤1 cup/wk | 1.00(reference) | Attained age, calendar period, |
| 1999 |  |  |  |  |  | 2-4 cups/wk | 1.03(0.78-1.37) | city, age at time of bombing, |
|  |  |  |  |  |  | ≥5 cups/wk | 1.19(0.93-1.52) | radiation dose |
|  |  |  |  |  |  |  |  |  |
| Michels et al^41^ | Sweden | 1987-1997 | 59,036 F | 1271 | Breast | ≤1 cup/wk | 1.00(reference) | Age, family history of breast cancer, |
| 2002 |  |  | Aged 40-76 y |  |  | 2-4 cups/wk | 0.81(0.54-1.22) | height, BMI, education, parity, |
|  |  |  |  |  |  | 1 cup/d | 0.99(0.75-1.28) | age at first birth, alcohol consumption, |
|  |  |  |  |  |  | 2-3 cups/d | 0.94(0.79-1.12) | total caloric intake |
|  |  |  |  |  |  | ≥4 cups/d | 0.94(0.75-1.28) |  |
| Suzuki et al^42^ | Japan | 1984-1997 | 35,004 F | 222 | Breast | Never | 1.00(reference) | Age, types of health insurance, |
| 2004 |  |  | Aged 40-64 y |  |  | Occasionally | 0.78(0.53-1.13) | age at menarche, menopausal status, |
|  |  |  |  |  |  | ≥1 cups/d | 0.81(0.55-1.18) | age at first birth, parity, BMI, smoking, |
|  |  |  |  |  |  |  |  | mother's history of breast cancer, alcohol |
|  |  |  |  |  |  |  |  | drinking, tea consumption frequencies |
| Hirvonen et al^43^ | France | 1994-2002 | 4,396 F | 95 | Breast | 0-111 mL/d | 1.00(reference) | Age, smoking, number of children, |
| 2006 |  |  | Aged 35-60 y |  |  | 112-252 mL/d | 1.07(0.64-1.79) | use of oral contraception, menopausal |
|  |  |  |  |  |  | ≥253 mL/d | 1.10(0.66-1.84) | status, family history of breast cancer, |
| Ganmaa et al^44^ | United | 1980-2002 | 85,987 F | 5272 | Breast | ＜1 cup/mo | 1.00(reference) | Age, BMI, physical activity, smoking status, |
| 2008 | States |  | Aged 33-55 y |  |  | 1 cup/m- | 1.01(0.92-1.12) | height, alcohol intake, tea intake, |
|  |  |  |  |  |  | 4.9 cups/wk |  | age at menarche and menopause, |
|  |  |  |  |  |  | 5 cups/wk- | 0.92(0.84-1.01) | menopausal status, use of hormone therapy, |
|  |  |  |  |  |  | 1.9 cups/d |  | parity age at first birth, weight change, |
|  |  |  |  |  |  | 2-3.9 cups/d | 0.93(0.85-1.02) | duration of postmenopausal hormone use, |
|  |  |  |  |  |  | ≥4 cups/d | 0.92(0.82-1.03) | family history of breast cancer |
|  |  |  |  |  |  |  |  |  |
| Pathy et al^45^ | Netherlands | 1993-2007 | 27,323 F | 681 | Breast | 0 cup/d | 0.74(0.52-1.05) | Age, smoking status, educational status, |
| 2009 |  |  | Aged 20-70 y |  |  | 0.1-1.0 cups/d | 1.00(reference) | alcohol intake, energy intake, BMI, |
|  |  |  |  |  |  | 1.1-2.0 cups/d | 0.93(0.74-1.17) | energy adjusted fiber and saturated |
|  |  |  |  |  |  | 2.1-3.0 cups/d | 0.90(0.68-1.19) | fat intake, ever use of oral contraceptives, |
|  |  |  |  |  |  | 3.1-5.0 cups/d | 0.83(0.65-1.06) | physical activity level, age at menarche, |
|  |  |  |  |  |  | ＞5 cups/d | 0.83(0.62-1.11) | presence of hypercholesterolemia, parity, |
|  |  |  |  |  |  |  |  | family history of breast cancer, cohort |
| Takezaki et al^46^ | Japan | 1985-1999 | 5,885 | 51 | Lung | Almost never | 1.00(reference) | Age, sex, |
| 2003 |  |  | Aged ≥30 y |  |  | occasionally | 1.22(0.63-2.34) | smoking, occupation |
|  |  |  |  |  |  | every day | 1.20(0.60-2.40) |  |
| Mills et al^47^ | United | 1976-1982 | 34,198 | 52 | Bladder | Never | 1.00(reference) | Age, sex, smoking |
| 1991 | States |  | 13,763 M |  |  | ＜1 cup/day | 0.98(0.41-2.31) |  |
|  |  |  | 20,435 F |  |  | 1 cup/day | 0.44(0.11-1.83) |  |
|  |  |  | Aged ≥25 y |  |  | ≥2 cups/day | 1.99(0.91-4.34) |  |
| Michaud et al^48^ | United | 1986-1996 | 47,909 M | 252 | Bladder | ＜1 cup/mo | 1.00(reference) | Geographic region, age, energy intake, |
| 1999 | States |  | Aged 40-75 y |  |  | 1 cup/mo- | 0.97(0.68-1.37) | pack-years of smoking, |
|  |  |  |  |  |  | 6 cups/wk |  | current smoking status, |
|  |  |  |  |  |  | 1-3 cups/day | 1.00(0.73-1.37) | intake of fruits and vegetables |
|  |  |  |  |  |  | ≥4 cups/day | 0.79(0.48-1.30) |  |
| Nagano et al^49^ | Japan | 1980-1993 | 38,540 | 89 | Bladder | 0/week | 1.00(reference) | Age, gender, |
| 2000 |  |  | 14,873 M |  |  | 1–4/week | 0.73(0.43-1.25) | radiation dose, smoking status, |
|  |  |  | 23,667 F |  |  | ≥5/week | 0.90(0.52-1.56) | education level, BMI, calendar time |
| Zeegers et al^50^ | Netherlands | 1986-1992 | 120,852 | 569 | Bladder | 0-＜2 cups/day | 1.00(reference) | Age, tea consumption, |
| 2001 |  |  | 58,279 M | 491 M |  | 2-＜3 cups/day | 0.87(0.41-1.33) | cigarette smoking |
|  |  |  | 62,573 F | 78 F |  | 3-＜4 cups/day | 1.26(0.64-1.88) |  |
|  |  |  | Aged 55-69 y |  |  | 4-＜5 cups/day | 0.60(0.28-0.92) |  |
|  |  |  |  |  |  | 5-＜6 cups/day | 0.60(0.26-0.93) |  |
|  |  |  |  |  |  | 6-＜7 cups/day | 1.71(1.07-2.84) |  |
|  |  |  |  |  |  | ≥7 cups/day | 1.95(1.23-3.23) |  |
| Tripathi et al^51^ | United | 1986-1998 | 37,459 F | 112 | Bladder | Never or | 1.00(reference) | Age |
| 2002 | States |  | Aged 55-69 y |  |  | ＜1 cup/mo |  |  |
|  |  |  |  |  |  | 1 cup/mo to | 1.00(0.56-1.79) |  |
|  |  |  |  |  |  | 5-6 cups/wk |  |  |
|  |  |  |  |  |  | 1 cup/d to | 1.00(0.60-1.64) |  |
|  |  |  |  |  |  | 2-3 cups/d |  |  |
|  |  |  |  |  |  | ≥4 cups/d | 1.59(0.95-2.68) |  |
| Hiatt et al^52^ | United | 1978-1984 | 122,894 | 49 | Pancreas | None | 1.0(reference) | Age, sex, ethnic origin, |
| 1988 | States |  |  |  |  | ＜1 cup/day | 0.4(0.1-4.3) | blood glucose levels, |
|  |  |  |  |  |  | 1-3 cups/day | 1.2(0.3-4.4) | consumption of alcohol, tea |
|  |  |  |  |  |  | ＞4 cups/d | 0.8(0.2-4.6) |  |
| Zheng et al^53^ | United | 1966-1986 | 17,633 M | 57 | Pancreas | ＜3 cups/day | 1.0(reference) | Age, smoking index, |
| 1993 | States |  | Aged ≥35 y |  |  | 3-4 cups/day | 0.6(0.3-1.2) | alcohol index |
|  |  |  |  |  |  | 5-6 cups/day | 0.7(0.4-1.6) |  |
|  |  |  |  |  |  | ≥7 cups/day | 0.9(0.3-2.4) |  |
| Shibata et al^54^ | United | 1981-1990 | 13,979 | 63 | Pancreas | ＜1 cup/day | 1.00(reference) | Sex, age, cigarette smoking |
| 1994 | States |  |  |  |  | 1 cup/day | 1.82(0.75-4.43) |  |
|  |  |  |  |  |  | 2-3 cups/day | 1.67(0.74-3.77) |  |
|  |  |  |  |  |  | ≥4 cups/day | 0.88(0.28-2.80) |  |
| Michaud et al^55^ | United | 1980-1998 | 136,593 | 288 | Pancreas | none | 1.00(reference) | Age in 5-year categories, |
| 2001 | States |  | 47,794 M | 130 M |  | ＜1 cup/d | 0.94(0.65-1.36) | pack-years of smoking, BMI, |
|  |  |  | Aged 40-75 y | 158 F |  | 1 cup/d | 0.60(0.38-0.94) | history of diabetes mellitus, |
|  |  |  | 88,799 F |  |  | 2-3 cup/d | 0.88(0.65-1.21) | history of cholecysectomy, |
|  |  |  | Aged 30-55 y |  |  | ＞3 cups/d | 0.62(0.27-1.43) | energy intake, period |
| Isaksson et al^56^ | Sweden | 1961-1997 | 21,884 | 131 | Pancreas | 0-2 cups/day | 1.00(reference) | Sex, age, |
| 2002 |  |  | 9,680 M |  |  | 3-6 cups/day | 0.91(0.60-1.38) | cigarette somking |
|  |  |  | 12,204 F |  |  | ≥7 cups/day | 0.39(0.17-0.89) |  |
|  |  |  | Aged 36-75 y |  |  |  |  |  |
| Lin et al^57^ | Japan | 1988-1997 | 99,527 | 225 | Pancreas | Nondrinkers | 1.00(reference) | Age, |
| 2002 |  |  | 44,646 M |  |  | 1-2 cups/month | 0.88(0.39-1.36) | cigarette smoking in pack-years |
|  |  |  | 54,881 F |  |  | 1-4 cups/week | 0.63(0.31-0.95) |  |
|  |  |  | Aged 40-79 y |  |  | 1 cup/day | 0.70(0.26-1.14) |  |
|  |  |  |  |  |  | ≥2 cups/day | 0.58(0.14-1.02) |  |
| Stolzenberg- Solomon et al^58^ | Finland | 1985-1997 | 27,111 M | 163 | Pancreas | ≤321.4 g/day | 1.00(reference) | Age, |
| 2002 |  |  | Aged 50-69 y |  |  | >321.4- | 1.48(0.89-2.46) | years of smoking |
|  |  |  |  |  |  | ≤450.0 g/day |  |  |
|  |  |  |  |  |  | >450.0- | 1.12(0.61-2.03) |  |
|  |  |  |  |  |  | ≤624.9 g/day |  |  |
|  |  |  |  |  |  | >624.9- | 1.72(1.01-2.86) |  |
|  |  |  |  |  |  | ≤878.6 g/day |  |  |
|  |  |  |  |  |  | >878.6 g/day | 0.95(0.54-1.68) |  |
| Luo et al^59^ | Japan | 1990-2003 | 102,137 | 233 | Pancreas | Rarely | 1.0(reference) | Age, BMI, frequency of sports, |
| 2007 |  |  | 48,783 M | 135 M |  | 1-2 cups/week | 1.0(0.7-1.4) | smoking status, alcohol intake, |
|  |  |  | 53,354 F | 98 F |  | 3-4 cups/week | 1.1(0.7-1.7) | history of diabetes, |
|  |  |  | Aged 40-69 y |  |  | 1-2 cups/day | 0.9(0.6-1.3) | history of cholelithiasis, |
|  |  |  |  |  |  | ≥3 cups/day | 0.8(0.4-1.3) | study area, tea consumption |
| Hsing et al^60^ | United | 1966-1986 | 17,633 M | 149 | Prostate | ＜3 cups/day | 1.0(reference) | Age, smoking index, |
| 1990 | States |  | Aged ≥35 y |  |  | 3-4 cups/day | 0.8(0.6-1.2) | alcohol index |
|  |  |  |  |  |  | ≥5 cups/day | 1.0(0.6-1.6) |  |
| Ellison et al^61^ | Canada | 1970-1993 | 3,400 M | 145 | Prostate | 0 | 1.00(reference) | Five-year age group, |
| 2000 |  |  | Aged 50-84 y |  |  | ＞0-250 ml/d | 1.14(0.66-1.97) | wine consumption |
|  |  |  |  |  |  | ＞250-500 ml/d | 1.42(0.80-2.52) |  |
|  |  |  |  |  |  | ＞500-750 ml/d | 1.35(0.75-2.43) |  |
|  |  |  |  |  |  | ＞750 ml/d | 1.42(0.77-2.61) |  |
| Washio et al^62^ | Japan | 1988-1999 | 114,517 | 33 | Kidney | None | 1.00(reference) | Age, sex |
| 2005 |  |  | 47,997 M |  |  | ≤2 cups/day | 1.25(0.67-2.33) |  |
|  |  |  | 66,520 F |  |  | ≥3 cup/day | 2.69(0.89-8.10) |  |
|  |  |  | Aged ≥40 y |  |  |  |  |  |
| Lee et al^63^ | United | 1980-2000 | 136,587 | 246 | Kidney | ＜1/mo | 1.00(reference) | BMI, history of hypertension, |
| 2006 | States |  | 47,828 M | 114 M |  | 1/mo-＜1/d | 0.91(0.62-1.34) | smoking status, alcohol intake, |
|  |  |  | Aged 40-75 y | 132 F |  | 1-＜3/d | 0.85(0.60-1.21) | multivitamin use (for men) |
|  |  |  | 88,759 F |  |  | ≥3/d | 0.84(0.54-1.30) | parity, history of diabetes (for women) |
|  |  |  | Aged 30-55 y |  |  |  |  |  |
| Abel et al^64^ | United | 1993-1998 | 77,375 F | 7,478 | Nonmelanoma | 0 cup/day | 1.00(reference) | Age at screening, alcohol consumption, |
| 2007 | States |  | Aged 50-79 y |  |  | 1 cup/day | 0.96(0.89-1.03) | income, education, β-carotene intake, |
|  |  |  |  |  |  | 2-3 cups/day | 0.87(0.81-0.92) | BMI, region of residence by latitude, |
|  |  |  |  |  |  | 4-5 cups/day | 0.83(0.75-0.92) | menopausal hormone therapy, smoking |
|  |  |  |  |  |  | ≥6 cups/day | 0.70(0.60-0.88) |  |
| Veierød et al^65^ | Norway | 1977-1992 | 50,757 | 108 | Melanoma | ≤2 cups/day | 1.00(reference) | County of residence, |
| 1997 |  |  | 25,708 M | 47 M |  | 3-4 cups/day | 0.63(0.23-1.03) | age at inclusion, attained age |
|  |  |  | 25,049 F | 61 F |  | 5-6 cups/day | 0.42(0.12-0.72) |  |
|  |  |  | Aged 16-56 y |  |  | ≥7 cups/day | 0.43(0.09-0.78) |  |
| Ma et al^66^ | United | 1995-2003 | 491,163 | 338 | Leukemia | Never | 1.00(reference) | Age, sex, smoking status, |
| 2010 | States |  | 292,724 M | 242 M |  | >0 and | 0.67(0.47-0.96) | total energy intake |
|  |  |  | 198,439 F | 96 F |  | <500 g/day |  |  |
|  |  |  |  |  |  | ≥500 and | 0.62(0.44-0.88) |  |
|  |  |  |  |  |  | <1000 g/day |  |  |
|  |  |  |  |  |  | ≥1000 g/day | 0.62(0.44-0.88) |  |
| Snowdon et al^67^ | United | 1960-1980 | 23,912 | 1159 | Bladder,Pancreas, | ＜1 cup/day | 1.00(reference) | Age, sex |
| 1984 | States |  | Aged ≥30 y |  | Colon,Breast, | 1 cup/day | 1.17(0.97-1.42) |  |
|  |  |  |  |  | Ovary,Others | ≥2 cups/day | 0.96(0.82-1.13) |  |
| Jacobsen et al^68^ | Norway | 1967-1978 | 16,555 | 1498 | Buccal cavity,Pharynx, | ≤2 cups/day | 1.00(reference) | Age, sex, residence |
| 1986 |  |  | 13,664 M |  | Esophagus,Stomach, | 3-4 cups/day | 1.05(0.92-1.19) | (Men for cigarette smoking) |
|  |  |  | 2,891 F |  | Colon,Rectum, | 5-6 cups/day | 0.87(0.75-1.01) |  |
|  |  |  |  |  | Pancreas,Larynx, | ≥7 cups/day | 0.88(0.73-1.06) |  |
|  |  |  |  |  | Trachea,Bronchus, |  |  |  |
|  |  |  |  |  | Lung,Breast,Cervix, |  |  |  |
|  |  |  |  |  | Corpus,Ovary,Kidney, |  |  |  |
|  |  |  |  |  | Prostate gland, |  |  |  |
|  |  |  |  |  | Bladder,Melanoma, |  |  |  |
|  |  |  |  |  | Nonmelanoma,Lymphoma, |  |  |  |
|  |  |  |  |  | Multiple myeloma, |  |  |  |
|  |  |  |  |  | Leukemia |  |  |  |
| Nomura et al^69^ | Japan | 1965-1983 | 7,355 | 672 | Lung,Colon, | 0 cup/day | 1.00(reference) | Age, year of smoking, |
| 1986 |  |  |  |  | Prostate,Stomach, | 1-2 cups/day | 0.66(0.53-0.83) | smoking status at exam, |
|  |  |  |  |  | Rectum,Bladder, | 3-4 cups/day | 0.78(0.62-0.98) | past smoking status, |
|  |  |  |  |  | Pancreas,Others | ≥5 cups/day | 0.86(0.68-1.08) | number of cigarettes smoked per day, |
| Stensvold et al^70^ | Norway | 1977-1990 | 42,973 | 1,519 | Buccal cavity,Pharyn, | ≤2 cups/day | 1.00(reference) | Age, cigarette smoking, |
| 1994 |  |  | 21,735 M | 671 M | Stomach,Colon,Rectum, | 3-4 cups/day | 0.91(0.76-1.09) | county of residence |
|  |  |  | 21,238 F | 848 F | Pancreas,Lung, | 5-6 cups/day | 0.92(0.76-1.09) |  |
|  |  |  | Aged 35-54 y |  | Prostate, Kidney, | ≥7 cups/day | 0.99(0.82-1.19) |  |
|  |  |  |  |  | Breast,Bladder, |  |  |  |
|  |  |  |  |  | Melanoma,Cervix uteri, | |  |  |
|  |  |  |  |  | Corpus uteri,Ovaries, |  |  |  |
|  |  |  |  |  | Endocrine glands, |  |  |  |
|  |  |  |  |  | Hodgkin's/non- |  |  |  |
|  |  |  |  |  | Hodgkin's lymphomas |  |  |  |
| Khan et al^71^ | Japan | 1984-2002 | 3,158 | 244 | Lung,Stomach, | ≤several | 1.00(reference) | Age, sex, health education, |
| 2004 |  |  |  |  |  | times/month |  | health examination, |
|  |  |  | 1,524 M | 155 M | Colorectal,Pancreas, | ≥several | 0.72(0.49-0.94) | health status, smoking |
|  |  |  |  |  |  | times/week |  |  |
|  |  |  | 1,634 F | 89 F | Others |  |  |  |
|  |  |  | Aged ≥40 y |  |  |  |  |  |
| Naganuma et al^72^ | Japan | 1990-2003 | 38,679 | 157 | Oral,Pharyngeal | never | 1.00(reference) | Age, sex, BMI, alcohol consumption, |
| 2008 |  |  | 18,858 M | 135 M | Esophageal | occasionally | 0.54(0.37-0.80) | cigarette smoking, green tea consumption, |
|  |  |  | 19,821 F | 22 F |  | ≥1 cup/d | 0.51(0.33-0.77) | consumption of vegetables and fruits |
|  |  |  | Aged 40-64 y |  |  |  |  |  |
| Zheng et al^73^ | United | 1986-1993 | 35,369 F | 1899 | Digestive tract | Never/monthly | 1.00(reference) | Age, education, smoking status, |
| 1996 | States |  | Aged 55-69 y |  | Urinary tract | Weekly- | 0.95(0.87-1.03) | pack-years of smoking, physical activity, |
|  |  |  |  |  | Ovary, Breast | 3 cups/day |  | all fruit and vegetable intake, |
|  |  |  |  |  |  | ≥4 cups/d | 0.97(0.85-1.11) | total energy intake, waist/hip ratio, |
|  |  |  |  |  |  |  | 0.96(0.89-1.02) | family history of cancer, |
|  |  |  |  |  |  |  |  | prior history of blood transfusion |
