# Supplementary material for: Coffee consumption and risk of cancers: a meta-analysis of cohort studies
Source: BMC Cancer. 2011 Mar 15;11:96. doi: 10.1186/1471-2407-11-96 (PMC3066123; doi:10.1186/1471-2407-11-96)
Supplement: Additional file 2 — Table S2. The summary RR for various cancer sites or different geographic regions and incremental estimates for 1 cup/day increment of coffee consumption. [file 1471-2407-11-96-S2.DOC]

Table S2 The summary RR for various cancer sites or different geographic regions and incremental estimates for 1 cup/day increment of coffee consumption.

| cancer sites |  | summary RR and corresponding | Heterogeneity test | | | RR for 1 cup/day |
| --- | --- | --- | --- | --- | --- | --- |
| /regions |  | 95% CI for cancer | Q | P | I^2^ (%) | increment of coffee |
| 40 cohorts | Total | 0.87(0.82-0.92) | 178.1 | <0.001 | 78.1 | 0.97(0.96-0.98) |
|  | M | 0.88(0.78-0.98) | 118.27 | <0.001 | 84.8 |  |
|  | F | 0.87(0.82-0.93) | 90.61 | <0.001 | 75.7 |  |
|  | Low to moderate consumption | 0.89(0.84-0.93) | 95.78 | <0.001 | 61.4 |  |
|  | High consumption | 0.82(0.74-0.89) | 114.71 | <0.001 | 67.7 |  |
| Bladder | Total | 0.83(0.73-0.94) | 13.18 | 0.106 | 39.3 | 1.03(0.99-1.06) |
| 9 cohorts | M | 0.78(0.58-0.98) | 9.24 | 0.055 | 56.7 |  |
|  | F | 0.77(0.52-1.01) | 8.8 | 0.032 | 65.9 |  |
|  | Low to moderate consumption | 0.79(0.67-0.91) | 11.75 | 0.163 | 31.9 |  |
|  | High consumption | 0.93(0.73-1.13) | 6.73 | 0.566 | 0 |  |
| Breast | Total | 0.94(0.91-0.98) | 14.02 | 0.172 | 28.7 | 0.99(0.97-1.00) |
| 11 cohorts | Low to moderate consumption | 0.94(0.89-0.99) | 8.3 | 0.6 | 0 |  |
|  | High consumption | 0.94(0.89-1.00) | 6.94 | 0.731 | 0 |  |
| Colorectum | Total | 0.89(0.80-0.97) | 56.58 | <0.001 | 75.3 | 0.99(0.97-1.01) |
| 15 cohorts | M | 0.87(0.73-1.00) | 27.27 | 0.001 | 67 |  |
|  | F | 0.95(0.88-1.03) | 11.35 | 0.183 | 29.5 |  |
|  | Colon | 0.93(0.84-1.01) | 15.59 | 0.076 | 42.3 |  |
|  | Rectum | 0.89(0.73-1.05) | 26.48 | 0.002 | 66 |  |
|  | Low to moderate consumption | 0.93(0.82-1.04) | 41.31 | <0.001 | 71 |  |
|  | High consumption | 0.88(0.77-0.99) | 21.11 | 0.049 | 43.2 |  |
| Endometrium | Total | 0.74(0.63-0.84) | 3.47 | 0.325 | 13.5 | 0.93(0.89-0.98) |
| 4 cohorts | Low to moderate consumption | 0.77(0.64-0.90) | 1.91 | 0.592 | 0 |  |
|  | High consumption | 0.68(0.51-0.85) | 3.04 | 0.386 | 1.3 |  |
| Esophagus | Total | 0.55(0.37-0.74) | 0.5 | 0.478 | 0 |  |
| 2 cohorts | Low to moderate consumption | 0.55(0.29-0.80) | 0.1 | 0.755 | 0 |  |
|  | High consumption | 0.58(0.29-0.87) | 0.28 | 0.596 | 0 |  |
| Stomach | Total | 1.00(0.77-1.24) | 20.96 | 0.004 | 66.6 | 1.13(1.03-1.24) |
| 8 cohorts | M | 0.88(0.69-1.06) | 6.77 | 0.239 | 26.1 |  |
|  | F | 1.05(0.40-1.71) | 17.58 | 0.001 | 77.3 |  |
|  | Low to moderate consumption | 0.97(0.71-1.22) | 9.98 | 0.076 | 49.9 |  |
|  | High consumption | 0.89(0.71-1.07) | 7.32 | 0.198 | 31.7 |  |
| Leukemia | Total | 0.64(0.51-0.77) | 1.44 | 0.231 | 30.4 |  |
| 2 cohorts | Low to moderate consumption | 0.65(0.49-0.82) | 1.14 | 0.285 | 12.7 |  |
|  | High consumption | 0.63(0.41-0.84) | 0.27 | 0.601 | 0 |  |
| Hepatocellular | Total | 0.54(0.46-0.61) | 5.64 | 0.228 | 29.1 | 0.83(0.78-0.87) |
| 5 cohorts | M | 0.53(0.38-0.67) | 8.24 | 0.041 | 63.6 |  |
|  | F | 0.59(0.41-0.77) | 0.11 | 0.991 | 0 |  |
|  | Low to moderate consumption | 0.63(0.53-0.72) | 3.63 | 0.459 | 0 |  |
|  | High consumption | 0.42(0.31-0.53) | 2.23 | 0.693 | 0 |  |
|  | with hepatocellular disease | 0.61(0.46-0.76) | 1.58 | 0.454 | 0 |  |
|  | without hepatocellular disease | 0.60(0.44-0.76) | 2.44 | 0.295 | 18.1 |  |
| Lung | Total | 1.17(0.92-1.42) | 3.18 | 0.529 | 0 |  |
| 5 cohorts | M | 1.10(0.78-1.42) | 5.52 | 0.137 | 45.7 |  |
|  | F | 1.00(0.42-1.59) | 0.3 | 0.585 | 0 |  |
|  | Low to moderate consumption | 1.22(0.89-1.56) | 0.56 | 0.906 | 0 |  |
|  | High consumption | 1.46(0.84-2.07) | 0.67 | 0.715 | 0 |  |
| Nonmelanoma | Total | 0.77(0.52-1.01) | 8.44 | 0.004 | 88.2 |  |
| 2 cohorts | F | 0.88(0.84-0.91) |  | 0.227 | 31.6 |  |
|  | Low to moderate consumption | 0.89(0.85-0.93) | 0.51 | 0.473 | 0 |  |
|  | High consumption | 0.55(0.22-0.88) | 5.09 | 0.024 | 80.4 |  |
| Buccal cavity and pharynx | Total | 0.49(0.29-0.70) | 2.71 | 0.258 | 26.1 |  |
| 3 cohorts | Low to moderate consumption | 0.60(0.30-0.90) | 1.55 | 0.461 | 0 |  |
|  | High consumption | 0.40(0.12-0.68) | 0.79 | 0.674 | 0 |  |
| Ovary | Total | 1.00(0.83-1.17) | 15.24 | 0.033 | 54.1 | 0.96(0.93-0.99) |
| 8 cohorts | Low to moderate consumption | 0.96(0.84-1.07) | 8.15 | 0.32 | 14.1 |  |
|  | High consumption | 0.93(0.75-1.11) | 7.06 | 0.423 | 0.8 |  |
| Pancreas | Total | 0.82(0.69-0.95) | 21.88 | 0.057 | 40.6 | 0.96(0.90-1.02) |
| 14 cohorts | M | 0.73(0.63-0.84) | 9.01 | 0.252 | 22.3 |  |
|  | F | 0.82(0.52-1.11) | 12.42 | 0.029 | 59.7 |  |
|  | Low to moderate consumption | 0.86(0.76-0.96) | 16.12 | 0.186 | 25.6 |  |
|  | High consumption | 0.68(0.51-0.84) | 7.82 | 0.729 | 0 |  |
| Prostate | Total | 0.79(0.61-0.98) | 9.33 | 0.053 | 57.1 |  |
| 5 cohorts | Low to moderate consumption | 0.76(0.63-0.89) | 7.19 | 0.126 | 44.4 |  |
|  | High consumption | 0.81(0.58-1.05) | 3.55 | 0.407 | 0 |  |
| Kidney | Total | 0.74(0.46-1.03) | 11.14 | 0.025 | 64.1 | 0.99(0.91-1.06) |
| 5 cohorts | M | 0.32(0.07-0.57) | 1.58 | 0.209 | 36.7 |  |
|  | F | 0.75(0.42-1.07) | 0.01 | 0.943 | 0 |  |
|  | Low to moderate consumption | 0.73(0.43-1.03) | 8.31 | 0.081 | 51.8 |  |
|  | High consumption | 0.74(0.48-1.00) | 3.46 | 0.484 | 0 |  |
| Asia | Total | 0.82(0.74-0.90) | 26.79 | 0.005 | 58.9 |  |
| 12 cohorts | M | 0.75(0.71-0.79) | 3.1 | 0.796 | 0 |  |
|  | F | 0.76(0.64-0.88) | 15.73 | 0.015 | 61.8 |  |
| Europe | Total | 0.85(0.72-0.98) | 82.37 | <0.001 | 87.9 |  |
| 13 cohorts | M | 0.81(0.60-1.02) | 68.9 | <0.001 | 92.7 |  |
|  | F | 0.83(0.67-0.98) | 73.98 | <0.001 | 90.5 |  |
| North America | Total | 0.92(0.86-0.98) | 46.9 | <0.001 | 70.2 |  |
| 15 cohorts | M | 1.05(0.80-1.30) | 14.83 | 0.005 | 73 |  |
|  | F | 0.95(0.88-1.02) | 19.26 | 0.002 | 74 |  |

**Note: Low to moderate coffee consumption:** Inoue et al^15^≥1 cup/week to ≤2 cups/day; Shimazu et al^16^ occasionally; Kurozawa et al^17^＜1 cup/day; Hu et al^18^ 2-5 cups/day; Shimazu et al^19^ 3 days/week to 2 cups/day; Friberg et al^20^ 2-3 cups/day; Larsson et al^21^ 2-3 cups/day; Silvera et al^22^ 0-3 cups/day; Steevens et al^23^ 2-＜5 cups/day; Tworoger et al^24^ 1 cup/month-2 cups/day; Galanis et al^26^ 1 cup/day; Tsubono et al^27^ occasionally; Larsson et al^28^ 2-3 cups/day; Wu et al^29^ 2-3 cups/day; Hartman et al^31^ >4 cups/d to ≤6 cups/day; Terry et al^32^ 2-3 cups/day; Michels et al^33^ 1 cup/month-2 cups/day; Larsson et al^34^ 2-3 cups/day; Oba et al^35^ 1 cup/month to <1 cup/day; Naganuma et al^36^ occasionally; Lee et al^37^≥1 cup/week to ≤2 cups/day; Peterson et al^38^ 1 cup/day; Hoyer et al^39^ 3-6 cups/day; Key et al^40^ 2-4 cups/week; Michels et al^41^ 2 cups/week to 3 cups/day; Suzuki et al^42^ occasionally; Hirvonen et al^43^ 112-252 mL/day; Ganmaa et al^44^ 1 cup/month-2 cups/day; Pathy et al^45^ 1.1-5.0 cups/day; Mills et al^47^≤1 cup/day; Michaud et al^48^ 1 cup/month-3 cups/day; Nagano et al^49^ 1–4/week; Zeegers et al^50^ 2-＜5 cups/day; Tripathi et al^51^ 1 cup/month-3 cups/day; Hiatt et al^52^＜3 cups/day; Zheng et al^53^ 3-6 cups/day; Shibata et al^54^ 1-3 cups/day; Michaud et al^55^ 1 cup/month-3 cups/day; Isaksson et al^56^ 3-6 cups/day; Lin et al^57^＜1 cup/day; Stolzenberg-Solomon et al^58^ >321.4 g to ≤878.6 g/day; Luo et al^59^≥1 cup/week to ≤2 cups/day; Hsing et al^60^ 3-4 cups/day; Ellison et al^61^＞0 ml/day to ≤750 ml/day; Washio et al^62^ ≤2 cups/day; Lee et al^63^ 1 cup/month to＜3 cups/day; Abel et al^64^ 1-5 cups/day; Veierød et al^65^ 3-6 cups/day; Ma et al^66^ >0 to <1000 g/day; Snowdon et al^67^ 1 cup/day; Jacobsen et al^68^ 3-6 cups/day; Nomura et al^69^ 1-4 cups/day; Stensvold et al^70^ 3-6 cups/day; Naganuma et al^72^ occasionally; Zheng et al^73^ weekly to 3 cups/day.

**High coffee consumption:** Inoue et al^15^≥3 cups/d; Shimazu et al^16^≥1 cup/day; Kurozawa et al^17^≥1 cup/day; Hu et al^18^≥6 cups/day; Shimazu et al^19^≥3 cups/day; Friberg et al^20^≥4 cups/day; Larsson et al^21^≥4 cups/day; Silvera et al^22^≥4 cups/day; Steevens et al^23^≥5 cups/day; Tworoger et al^24^≥3 cups/day; Galanis et al^26^≥2 cup/day; Tsubono et al^27^≥1 cup/day; Larsson et al^28^≥4 cups/day; Wu et al^29^≥4 cups/day; Hartman et al^31^ >6 cups/day; Terry et al^32^≥4 cups/day; Michels et al^33^≥3 cups/day; Larsson et al^34^≥4 cups/day; Oba et al^35^≥1 cup/day; Naganuma et al^36^≥1 cup/day; Lee et al^37^≥3 cups/day; Peterson et al^38^≥2 cups/day; Hoyer et al^39^≥7 cups/day; Key et al^40^≥5 cups/week; Michels et al^41^≥4 cups/day; Suzuki et al^42^≥1 cups/day; Hirvonen et al^43^≥253 mL/day; Ganmaa et al^44^≥3 cups/day; Pathy et al^45^＞5 cups/day; Mills et al^47^≥2 cups/day; Michaud et al^48^≥4 cups/day; Nagano et al^49^≥5/week; Zeegers et al^50^≥5 cups/day; Tripathi et al^51^≥4 cups/day; Hiatt et al^52^＞4 cups/day; Zheng et al^53^≥7 cups/day; Shibata et al^54^≥4 cups/day; Michaud et al^55^≥3 cups/day; Isaksson et al^56^≥7 cups/day; Lin et al^57^≥1 cup/day; Stolzenberg-Solomon et al^58^ >878.6 g/day; Luo et al^59^≥3 cups/day; Hsing et al^60^≥5 cups/day; Ellison et al^61^＞750 ml/day; Washio et al^62^≥3 cups/day; Lee et al^63^≥3 cups/day; Abel et al^64^≥6 cups/day; Veierød et al^65^≥7 cups/day; Ma et al^66^≥1000 g/day; Snowdon et al^67^≥2 cups/day; Jacobsen et al^68^≥7 cups/day; Nomura et al^69^≥5 cups/day; Stensvold et al^70^≥7 cups/day; Naganuma et al^72^≥1 cup/day; Zheng et al^73^≥4 cups/day.
